# Relationships among weight stigma, eating behaviors and stress in adolescents in Wuhan, China

**DOI:** 10.1186/s41256-020-00138-3

**Published:** 2020-03-07

**Authors:** Zhanxia Wang, Bowen Wang, Yiluan Hu, Lei Cheng, Siqi Zhang, Yanan Chen, Rui Li

**Affiliations:** 1 Department of Healthcare Management, School of Health Sciences, Wuhan University, Wuhan, 430071 China; 2 Global Health Institute, School of Health Sciences, Wuhan University, Wuhan, 430071 China

**Keywords:** Weight stigma, Eating behaviors, Stress, Adolescents

## Abstract

**Background:**

The purpose of this study was to examine the relationships among weight stigma, eating behaviors, and stress, as well as to analyze the effect of stress in mediating the association between weight stigma and eating behaviors.

**Methods:**

The study involved 1818 adolescents between 14 to 19 years of age and was conducted in Wuhan, China in 2019. Weight stigma, eating behaviors (cognitive restraint, uncontrolled eating, and emotional eating), and stress were assessed by self-report questionnaires. Hierarchical linear regression analyses were used to examine the predictors of cognitive restraint, uncontrolled eating, and emotional eating; the serial mediation models analyses were conducted to analyze the effect of stress in mediating the association between weight stigma and eating behaviors for the whole non-overweight (normal and underweight) and overweight or obese participants, respectively.

**Results:**

Hierarchical linear regression analyses showed that experiences of weight stigma significantly predicted uncontrolled eating and emotional eating regardless of body mass index (BMI) (non-overweight adolescents: uncontrolled eating: β [SE] = 0.161 [0.017]; emotional eating: β [SE] = 0.199 [0.008], *p* < 0.05; overweight or obese adolescents: uncontrolled eating: β [SE] = 0.286 [0.030]; emotional eating: β [SE] = 0.267 [0.014], *p* < 0.05); experiences of weight stigma significantly predicted cognitive restraint among non-overweight adolescents (β [SE] = 0.204 [0.013], *p* < 0.05). Mediation analyses showed that stress mediated the associations between weight stigma and uncontrolled eating and emotional eating among non-overweight adolescents (uncontrolled eating: indirect effect coefficient = 0.0352, 95% CI = 0.0241, 0.0478; emotional eating: indirect effect coefficient = 0.0133, 95% CI = 0.0085, 0.0186).

**Conclusions:**

These findings suggest that non-overweight individuals can still experience weight stigma and its associated negative consequences; the relationship between weight stigma and eating behaviors is modulated by weight status; stress mediated the associations between weight stigma and uncontrolled and emotional eating among non-overweight adolescents.

## Background

Weight stigma refers to an identity threat in response to negative weight-related biases, stereotypes, and prejudices due to overweight or obesity; it can be subtly or openly manifested [1, 2]. It is widespread across the world [3] and occurs in different groups, regardless of age and body weight status [2, 4–7]. However, studies have shown that weight stigma is more prevalent among overweight or obese individuals [8]. Over the past few decades, the rates of weight-based stigma have risen as the rates of obesity rise [9]. Experiencing weight stigma can be detrimental to both psychological and physical health [2, 10]. Negatives outcomes include depression [11], poor self-esteem [12], social isolation [13], unhealthy eating behaviors [14, 15], decreased physical activity [16], and increased weight gain [17].

Weight stigma among youth is most often experienced as victimization, teasing, and bullying [18]. It emerges as young as 3 years old when overweight children are described as “mean”, “stupid”, “lazy”, and “ugly” [19]. It has been demonstrated that adolescents are teased or bullied at school due primarily to their body weight status [20]. Recently, Bucchianeri et al. [21] conducted a study in a sample of racially diverse adolescents and found that weight-based harassment was the most prevalent form of harassment among girls and the second-most common form of harassment among boys. Similarly, 29% of adolescents from two American high schools in 2009 reported weight victimization, of which a substantial proportion (65%) having a body mass index (BMI) in the normal range [22]. A study of adolescents enrolling in weight loss camps found that 71% reported experiencing victimization due to their weight at school in the past year, and more than one-third indicated that the victimization had persisted for > 5 years [5]. And another study conducted in Japan showed that 23.9% of adolescents reported being teased because of their body; students who were overweight, of an upper-normal weight status, and perceived themselves as “fat” were at a greater risk of being teased [23]. These studies suggest that adolescents are vulnerable to weight stigma and its negative consequences in different settings.

The relationships among weight stigma, eating behaviors, and stress in adolescents are yet to be defined. Tomiyama [24] recently proposed that experiencing weight stigma could induce stress, leading to changes in cognition, behaviors (eating, physical activity, sleep), physiology, and biochemistry. Consequently, the vulnerability to obesity and weight stigma increased. O’Brien [25] found that weight bias internalization and psychological distress (depression, anxiety, and stress) mediated the relationship between weight stigma and disordered eating behavior after accounting for age, gender, and weight status among university students in Australia. Similarly, Salwen [26] reported that emotional responses to weight stigma fully mediated the relationships between weight stigma and emotional eating, binge eating, and eating at night among American undergraduate students.

The present study aims to examine the relationships among weight stigma, eating behaviors, and stress in Chinese adolescents. We hypothesized that weight stigma and stress could affect eating behaviors and that the effect of weight stigma on eating behaviors was at least partially mediated by stress.

## Methods

### Study population

A cross-sectional survey was conducted at the first high school of Jiangxia District in Wuhan, Hubei, China, during the late spring/early summer of 2019. The study was conducted in accordance with the Declaration of Helsinki, and the protocol was approved by the Ethics Committee of Wuhan University (Project Identification Code 2019YF2056). All adolescents (*n* = 2395) enrolled in grades 10–12 were invited to participate in the study. All students signed an consent form before the distribution of questionnaires to confirm their willingness to participate. Four hundred ten students were excluded before entering the study due to absence or refusing to participate in this study. In total, 1985 students agreed and completed the survey, with a response rate of 82.9%.

### Study design

Participants completed self-reported questionnaires in their classrooms under the direction of trained research staff to assess weight stigma, eating behaviors (cognitive restraint, uncontrolled eating, and emotional eating), and stress. The scales included the Perception of Teasing Scale (POTS), the Three-Factor Eating Questionnaire-Revised 18-item version (TFEQ-R18), and the 10-item Perceived Stress Scale (PSS-10).

### Measures

#### Body mass index (BMI)

BMI (kg/m^2^) was calculated from self-reported weight and height. The Chinese age- and gender-specific BMI cut-off points for children developed by the Group of China Obesity Task Force (COTF) [27] were used to classify participants as normal-weight, overweight, and obese. These cut-off points corresponded to approximately the 85th (overweight) percentile in a large national sample of children aged 7–18 years. In this study, adolescents were categorized as non-overweight (normal and underweight) and overweight or obese. An individual is considered overweight or obese if he or she falls in the 85th percentile; the weight status of an individual aged over 18 years was classified using the Chinese BMI cut-points for overweight or obese (BMI ≥ 24 kg/m^2^).

#### Weight stigma

The POTS is a reliable and valid measure of weight-related teasing experiences [28]. The scale includes 12-items which address: 1) the frequency of weight stigma, and 2) the extent to which stigmatizing events upset the individual. Subjects were asked to rate the frequency of being teased for fatness on a 5-point Likert scale from “Never” (1) to “Very often” (5) (6 items). The subjects were also asked to rate the effect of teasing (i.e. how upset they were) on a 5-point Likert scale from “Not upset” (1) to “Very upset” (5). A stigma-total score was calculated by summing the stigma frequency and upset scale scores. The stigma-total score ranges from 6 to 60, with higher scores representing greater weight stigma. Participants with stigma-total scores higher than 6 are considered to have experienced at least one incident of weight stigma. The scale has been found to have an internal consistency reliability of 0.930 for the stigma-total score in obese Chinese adolescent girls [29]. Cronbach’s alpha for the stigma frequency and upset items in the present sample were 0.893 and 0.957, respectively. The internal consistency reliability (Cronbach’s alpha) of the POTS in the present study was 0.920.

#### Eating behaviors

The TFEQ-R18 refers to current dietary practice and measures three domains of eating behaviors: nine items for uncontrolled eating, six for cognitive restraint, and three for emotional eating. Uncontrolled eating is an uncontrollable overeating behavior. Cognitive restraint refers to a tendency to consciously restrict food intake, but not necessarily equal to diet. Emotional eating refers to a tendency to consume food after negative emotions [30]. Uncontrolled eating, cognitive restraint, and emotional eating score range from 9 to 36, 5 to 25, and 3 to 12, respectively. A higher score indicates a stronger tendency of eating disturbance. The Chinese version of the TFEQ-R18 has been used in another study which was conducted among undergraduate students in Hong Kong [31]. The internal consistency reliability (Cronbach’s alpha) of the TFEQ-R18 in the present study was 0.823.

#### Stress

The PSS-10 asks respondents to stipulate the degree to which situations in their lives can be appraised as stressful over the past month [32]. The scale include six negative items that assess lack of control and negative affective reactions, and four positive items that measure the degree of ability to cope with existing stressors [32]. Each item is rated on a five-point Likert scale from 0 = “never” to 4 = “very often”. Total scores are obtained by reversing responses (e.g., 0 = 4, 1 = 3, 2 = 2, 3 = 1, 4 = 0) to the four positive items (items 4, 5, 7, &8) and then summing across all scale items. A higher score indicates a higher level of perceived stress. The scale has been found to have an internal consistency reliability of 0.85 in Chinese university students (Chinese version) [33]. The internal consistency (Cronbach’s alpha) of the PSS-10 in the present study was 0.825.

### Statistical analyses

First, descriptive analyses were conducted for all demographic data and questionnaire scores. T-test analyses were used to examine group differences between non-overweight and overweight or obese participants in boys and girls, respectively. Second, Pearson’s coefficients (r) were calculated, and the coefficients for variables were reported separately for the whole non-overweight and overweight or obese participants. Third, all the variables were inserted in hierarchical linear regression analyses to examine the predictors of cognitive restraint, uncontrolled eating, and emotional eating for the whole non-overweight and overweight or obese participants, respectively. Model 1 controlled for adolescents’ age and sex; model 2 included adolescents’ weight stigma; and model 3 included adolescents’ stress. Finally, to examine our hypothesis that weight stigma and stress could affect eating behaviors and that the effect of weight stigma on eating behaviors was at least partially mediated by stress, we utilised the serial mediation model in the PROCESS macro (Model 4) [34] for SPSS. All statistical analyses were performed using SPSS 22.0 [35]. *P* values less than 0.05 were considered statistically significant.

## Results

### Descriptive statistics for all study variables

Descriptive statistics for all the study variables are presented in Table 1. A sample of 1985 students agreed to participate, of which 167 were excluded due to incomplete questionnaires, missing height and weight information, and being over 19 years old. The sample (*n* = 1818) included 987 (54%) boys aged 16.5 years (SD = 0.98), with mean BMI of 21.2 kg/m^2^ (SD = 3.52). Among non-overweight boys, 23.9% experienced weight stigma; and that number for girls is 52.5%.

**Table 1:** Descriptive statistic for all study variables

	Boy (*n* = 987)				Girl (*n* = 831)			
	Mean (SD) Non-overweight(*n* = 742)	Mean (SD) Overweight or Obese (*n* = 245)	T-Value	*P*-Value*	Mean (SD) Non-overweight (*n* = 745)	Mean (SD) Overweight or Obese (*n* = 86)	T-value	*P*-Value*
Age	16.5 ± 1.0	16.5 ± 0.9	0.779	0.436	16.4 ± 1.0	16.4 ± 0.9	−0.038	0.969
BMI	20.2 ± 2.0	26.6 ± 2.7	−33.572	0.000	19.9 ± 1.8	26.0 ± 2.1	−25.508	0.000
Stigma-total	9.2 ± 7.1	19.4 ± 10.7	−13.913	0.000	12.8 ± 9.1	22.0 ± 10.9	−7.540	0.000
Cognitive restraint	11.2 ± 4.2	12.8 ± 3.8	−5.774	0.000	12.6 ± 3.8	12.7 ± 3.8	−0.353	0.724
Uncontrolled eating	18.0 ± 5.4	19.3 ± 6.1	−3.105	0.002	20.3 ± 5.3	20.7 ± 5.2	−0.724	0.469
Emotional eating	5.3 ± 2.4	6.0 ± 2.8	−3.668	0.000	6.4 ± 2.6	6.1 ± 2.7	1.004	0.316
Stress	19.9 ± 7.0	19.3 ± 6.8	1.363	0.173	20.9 ± 6.6	20.2 ± 7.5	0.930	0.353

*T-Test analysis of group differences between non-overweight and overweight or obese participants in boys and girls, respectively

### Correlations between all study variables

The correlations of measured variables are shown in Table 2. There were a number of significant correlations between the predictor variables and the eating behaviors (see Table 2). For non-overweight participants (see Table 2), stigma was strongly correlated with sex, cognitive restraint, uncontrolled eating, emotional eating, and stress. Stress was related to sex, uncontrolled eating, and emotional eating. Stigma was strongly correlated with cognitive restraint, uncontrolled eating, emotional eating, and stress both in girls and boys (see Additional file 1: Table S1).

**Table 2:** Correlations between all study variables

	1	2	3	4	5	6	7
**1. Sex**	–	−0.033	0.103	−0.011	0.108	0.007	0.058
**2. Age**	−0.066*	–	−0.066	0.070	−0.174**	− 0.038	−0.041
**3. Stigma**	0.212***	0.059*	–	0.015	0.335***	0.299***	0.313***
**4. Cognitive restraint**	0.174***	−0.037	0.224***	–	−0.175**	− 0.077	−0.034
**5. Uncontrolled eating**	0.210***	−0.084**	0.244***	0.027	–	0.618***	0.203***
**6. Emotional eating**	0.213***	0.016	0.278***	0.099***	0.625***	–	0.191***
**7. Perceived stress**	0.069**	0.018	0.254***	0.041	0.264***	0.235***	–

Correlations for overweight or obese participants (*n* = 331) are displayed above the diagonal, and non-overweight participants (*n* = 1487) are displayed below the diagonal

Categorical variables were dichotomised and coded as 1 and 2 to aid in interpretation. Sex (1 = boy, 2 = girl)

*p < 0.05, **p < 0.01, ***p < 0.001

For overweight participants, stigma was strongly correlated with uncontrolled eating, emotional eating, and stress. Stress was also strongly related to uncontrolled and emotional eating. Weight stigma of girls was less significantly related to uncontrolled eating and emotional eating than boys’ (see Additional file 1: Table S2).

### Predictors of cognitive restraint, uncontrolled eating, and emotional eating

#### Predictors of cognitive restraint, uncontrolled eating, and emotional eating for the non-overweight adolescents

Table 3 displays the results of the hierarchical regression analyses for the non-overweight group where each of the three eating behaviors was regressed onto the predictor variables.

**Table 3:** Hierarchical linear regression analyses for the non-overweight adolescents (*n* = 1487)

Variables	Model1			Model2			Model3		
	B	SE	β	B	SE	β	B	SE	β
Cognitive restraint									
Age	− 0.106	0.105	− 0.026	− 0.166	0.103	− 0.04	− 0.165	0.103	− 0.040
Sex	1.399	0.208	0.173***	1.048	0.209	0.129***	1.051	0.209	0.130***
Stigma				0.097	0.013	0.199***	0.099	0.013	0.204***
Stress							−0.011	0.015	−0.019
*R*^2^			0.031***			0.069***			0.069
Uncontrolled eating									
Age	−0.386	0.14	−0.07**	− 0.473	0.137	− 0.086**	−0.478	0.134	−0.087***
Sex	2.244	0.277	0.206***	1.734	0.277	0.159***	1.696	0.271	0.155***
Stigma				0.141	0.017	0.215***	0.106	0.017	0.161***
Stress							0.172	0.020	0.214***
*R*^2^			0.049***			0.093***			0.136***
Emotional eating									
Age	0.078	0.065	0.03	0.032	0.063	0.013	0.030	0.062	0.012
Sex	1.092	0.129	0.215***	0.825	0.128	0.163***	0.811	0.126	0.160***
Stigma				0.074	0.008	0.242***	0.061	0.008	0.199***
Stress							0.065	0.009	0.173***
*R*^2^			0.046***			0.102***			0.130***

*B* Unstandardized coefficients, *SE* Standard errors, *β* Standardized coefficients

**p < 0.01, ***p < 0.001

For cognitive restraint, the predictor variables except stress and age accounted for significantly variance, with the full model (Model 3) explaining 6.9% of the variance. Age and sex accounted for 3.1% of the variance in the initial model. Stigma accounted for an additional 3.8% of the variance in cognitive restraint scores. For uncontrolled eating, age and sex accounted for 4.9% of the variance in the initial model, with the entry of stigma accounting for an additional 4.4% of the variance. The association between stigma and uncontrolled eating was reduced following the entry of stress scores. Stress was a significant predictor in the final model, accounting for an additional 4.3% of the variance. A similar pattern of results was observed for emotional eating. The initial model containing age and sex accounted for 4.6% of the variance. In the second model, stigma was a significant predictor of emotional eating and accounted for another 5.6% of variance in eating scores. The association between stigma and emotional eating was reduced following the entry of stress scores. The entry of stress in a final model accounted for an additional 2.8% of the variance.

These findings suggest that, after controlling for a participant’s age and sex, among non-overweight adolescents, the experience of weight stigma and stress are associated with greater levels of uncontrolled eating and emotional eating, only weight stigma is associated with greater levels of cognitive restraint.

After controlling for a participant’s age, stress is associated with uncontrolled eating and emotional eating in boys and girls among non-overweight group (see Additional file 1: Tables S3 and S4).

#### Predictors of cognitive restraint, uncontrolled eating, and emotional eating for the overweight or obese adolescents

Table 4 displays the results of the hierarchical regression analyses for the overweight or obese group where each of the three eating behaviors was regressed onto the predictor variables.

**Table 4:** Hierarchical linear regression analyses for the overweight or obese adolescents (*n* = 331)

Variables	Model1			Model2			Model3		
	B	SE	β	B	SE	β	B	SE	β
Cognitive restraint									
Age	0.282	0.222	0.070	0.287	0.223	0.071	0.284	0.223	0.070
Sex	−0.073	0.473	−0.008	− 0.090	0.476	− 0.011	− 0.081	0.476	− 0.010
Stigma				0.007	0.019	0.020	0.011	0.020	0.026
Stress							−0.022	0.031	−0.040
*R*^2^			0.005			0.005			0.007
Uncontrolled eating									
Age	−1.084	0.343	−0.171**	−0.959	0.326	−0.151**	−0.946	0.325	−0.149**
Sex	1.378	0.73	0.102	0.944	0.696	0.07	0.908	0.693	0.067
Stigma				0.175	0.028	0.318***	0.157	0.030	0.286***
Stress							0.087	0.046	0.103
*R*^2^			0.041**			0.141***			0.150
Emotional eating									
Age	−0.113	0.166	−0.038	−0.057	0.159	−0.019	− 0.051	0.158	− 0.017
Sex	0.038	0.353	0.006	−0.156	0.339	−0.024	−0.174	0.338	−0.027
Stigma				0.078	0.014	0.301***	0.069	0.014	0.267***
Stress							0.043	0.022	0.109
*R*^2^			0.001			0.091***			0.101

*B* Unstandardized coefficients, *SE* Standard errors, *β* Standardized coefficients

**p < 0.01, ***p < 0.001

For cognitive restraint, all models were not significant (all *p* > 0.05). For uncontrolled eating, age and sex accounted for 4.1% of the variance in the initial model, with the entry of stigma accounting for an additional 10% of the variance. The association between stigma and uncontrolled eating was reduced following the entry of stress scores. The entry of stress accounted for an additional 0.9% of the variance and stress was not a significant predictor in the final model. The initial model containing age and sex accounted for 0.1% of the variance in the emotional eating. In the second model, stigma was a significant predictor of emotional eating and accounted for another 9% of variance in eating scores. The association between stigma and emotional eating was reduced following the entry of stress scores. The entry of stress accounted for an additional 1% of the variance. Only stigma-total was a significant predictor of emotional eating in the final model.

These findings suggest that, after controlling for a participant’s age and sex, the experience of weight stigma is significantly associated with greater levels of uncontrolled eating and emotional eating among overweight/obese adolescents. After controlling for a participant’s age, stress is associated with uncontrolled eating only in girls among overweight group, and weight stigma is not significantly associated with uncontrolled eating and emotional eating in girls, compared to boys (see Additional file 1: Tables S5 and S6).

### Mediation analyses for the associations of weight stigma on eating behaviors through stress

#### Mediation analyses for the non-overweight adolescents

Among non-overweight adolescents, in serial mediation analyses controlling for age and sex, the indirect path from stigma to eating behavior through stress was statistically significant for uncontrolled eating and emotional eating outcomes (see Table 5 for all path coefficients). The indirect path was not significant for cognitive restraint among non-overweight adolescents. For cognitive restraint, the indirect effect coefficient was − 0.0023, SE = 0.0035, 95% CI = − 0.0090, 0.0046; for uncontrolled eating, the indirect effect coefficient was 0.0352, SE = 0.0060, 95% CI = 0.0241, 0.0478; and for emotional eating, the indirect effect coefficient was 0.0133, SE = 0.0026, 95% CI = 0.0085, 0.0186.

**Table 5:** The serial mediation models in non-overweight adolescents (*n* = 1487)

Antecedent	Consequent							
		Stress				Cognitive restraint		
		coeff.	SE	*P*-Value		coeff.	SE	*P*-Value
Stigma	a_1_	0.2053	0.0211	0.0000***	c’	0.0994	0.0129	0.0000***
Stress		–	–	–	b_1_	−0.0111	0.0154	0.4708
Antecedent		Stress				Uncontrolled eating		
		coeff.	SE	*P*		coeff.	SE	*P*
Stigma	a_1_	0.2053	0.0211	0.0000***	c’	0.1060	0.0168	0.0000***
Stress		–	–	–	b_1_	0.1716	0.0200	0.0000***
Antecedent		Stress				Emotional eating		
		coeff.	SE	*P*		coeff.	SE	*p*
Stigma total	a_1_	0.2053	0.0211	0.0000***	c’	0.0607	0.0078	0.0000***
Stress		–	–	–	b_1_	0.0646	0.0093	0.0000***

*coeff.* Regression coefficients, *SE* Standard errors

***p < 0.001

Results indicate that a adolescent who has weight stigma experience is more likely to have uncontrolled and emotional eating via stress both in girls and boys among non-overweight group (see Additional file 1: Tables S7 and S8).

#### Mediation analyses for the overweight or obese adolescents

Among overweight or obese adolescents, in serial mediation analyses controlling for age and sex, the indirect path was non-significant for three eating behaviors (see Table 6 for all path coefficients). For cognitive restraint, the indirect effect coefficient was − 0.0044, SE = 0.0082, 95% CI = − 0.0208, 0.0119; for uncontrolled eating, the indirect effect coefficient was 0.0175, SE = 0.0137, 95% CI = − 0.0060, 0.0473; and for emotional eating, the indirect effect coefficient was 0.0087, SE = 0.0058, 95% CI = − 0.0014, 0.0210.

**Table 6:** The serial mediation models in overweight or obese adolescents (*n* = 331)

Antecedent	Consequent							
		Stress				Cognitive restraint		
		coeff.	SE	*P*-Value		coeff.	SE	*P*-Value
Stigma	a_1_	0.2008	0.0343	0.0000***	c’	0.0114	0.0204	0.5750
Stress		–	–	–	b_1_	−0.0217	0.0313	0.4880
Antecedent		Stress				Uncontrolled eating		
		coeff.	SE	*p*		coeff.	SE	*p*
Stigma	a_1_	0.2008	0.0343	0.0000***	c’	0.1570	0.0297	0.0000***
Stress		–	–	–	b_1_	0.0872	0.0455	0.0563
Antecedent		Stress				Emotional eating		
		coeff.	SE	*p*		coeff.	SE	*p*
Stigma total	a_1_	0.2008	0.0343	0.0000***	c’	0.0694	0.0144	0.0000***
Stress		–	–	–	b_1_	0.0435	0.0222	0.0506

*coeff.* Regression coefficients, *SE* Standard errors

***p < 0.001

Results indicate that compared to an overweight adolescents, a non-overweight adolescents who has weight stigma experience is more likely to have uncontrolled and emotional eating via stress.

Results also indicate that compared to a a boy, a girl who has weight stigma experience is more likely to have uncontrolled via stress among overweight group (see Additional file 1: Tables S9 and S10).

## Discussion

This study examined the predictive relationships of weight stigma, eating behaviors, and stress among non-overweight and overweight or obese adolescents in Wuhan, China. Experiences of weight stigma significantly predicted uncontrolled eating and emotional eating regardless of BMI in the present study. However, experiences of weight stigma significantly predicted cognitive restraint among non-overweight adolescents, not overweight or obese adolescents. Stress mediated the association between experiences of weight stigma and uncontrolled and emotional eating among non-overweight adolescents, but not overweight or obese adolescents.

The results also indicate that weight stigma not only works in individuals with overweight or obesity but also in non-overweight individuals. However, it is noteworthy that a similar but different pattern of associations was found for overweight and non-overweight participants, even though participants who were overweight experienced higher levels of weight stigma than non-overweight participants, and even though the magnitude of the association between weight stigma and the psychological and behavioral outcomes was somewhat greater for overweight participants. Accordingly, the findings are also consistent with other work [14, 31, 36] indicating that even individuals who were not classified as overweight or obese by BMI standards can still experience weight stigma and its associated negative consequences. Compared with overweight group, weight stigma is associated with cognitive restraint among adolescents who are in non-overweight group. A possible reason is that perceived pressure to be thin, thin-ideal internalization and thinness expectancies affect adolescents’ cognitions (e.g., body dissatisfaction) and behaviors (e.g., cognitive restraint) [37–39]. Similarly, a Canadian study reveals that being teased about weight was associated with dietary restraint in non-overweight people [13]. With the thinness pressures and value for thinness, non-overweight adolescents would consciously reduce their food intake to control weight and might try to prevent possible or already existed weight stigma even if they are not overweight. All individuals regardless of BMI who experienced weight stigma significantly predicted uncontrolled eating and emotional eating in the present study. In recent other studies, weight stigma has also been shown to encourage uncontrolled eating and emotional eating, which can undermine weight loss efforts and lead to weight gain [25, 40–42]. In addition, weight stigma was associated with stress in both non-overweight and overweight group. According to previous studies, weight stigma has also been linked to increased stress [43–46]. Although weight stigma was associated with uncontrolled and emotional eating among non-overweight group and overweight or obese group, individuals respond differently to mechanisms ranging from weight stigma to uncontrolled and emotional eating. Stress mediated the associations between weight stigma and uncontrolled and emotional eating only among non-overweight group not in overweight or obese group. One possible reason is that other types of negative emotions (e.g., anxiety) and variables (e.g., self-esteem) may significantly mediate the associations between weight stigma and eating behaviors in overweight or obese group, which can be examined in future study.

A number of strengths of the present study should be enumerated. First, the relatively large sample of adolescents and its diversity allowed for comparisons across weight. Second, the scales showed good internal consistency and there was some evidence of its validity. \Although the present study had a number of strengths, there are still some limitations. First, the data were collected as part of a self-report questionnaire, particularly weight and height. The main disadvantage of self-report questionnaires might be the possibility of providing invalid answers [47]. Second, the cross-sectional design is adopted in this study, and the direction of correlation between dependent variables and independent variables cannot be determined. Third, the convenience sampling method recruited in one high school might restrict the generalizability of our results. Lastly and importantly, R^2^ was relatively small in our regression models. Therefore, the results of our study might experience from low internal validity as we did not include sufficient confounding variables. Future research should include measured height and weight instead of just relying on self-reported height and weight. Future research should also use designs more appropriate for drawing causal conclusions (i.e., longitudinal designs).

## Conclusions

In conclusion, the relationship between weight stigma and eating behaviors is moderated by weight status, and that individuals who were not classified as overweight or obese by BMI standards can still experience weight stigma and its associated negative consequences. We identified weight stigma as prospectively associated with uncontrolled and emotional eating via the mediating mechanism of increased levels of stress among non-overweight adolescents. Further research is needed to identify how best to prevent weight stigma among adolescents.

## Supplementary information

**Additional file 1: Table S1.** Correlations between all study variables. Correlations for boys in non-overweight participants (*n* = 742) and girls in non-overweight participants (*n* = 745), respectively. **Table S2.** Correlations between all study variables. Correlations for boys in overweight or obese participants (*n* = 742) and girls in overweight or obese participants (*n* = 745), respectively. **Table S3.** Hierarchical linear regression analyses in boys in non-overweight participants (*n* = 742). **Table S4.** Hierarchical linear regression analyses in girls in non-overweight participants (*n* = 745). **Table S5.** Hierarchical linear regression analyses in boys in overweight or obese participants (*n* = 245). **Table S6.** Hierarchical linear regression analyses in girls in overweight or obese participants (*n* = 86). **Table S7.** The serial mediation models in boys in non-overweight participants (*n* = 742). **Table S8.** The serial mediation models in girls in non-overweight participants (*n* = 745). **Table S9.** The serial mediation models in boys in overweight or obese participants (*n* = 245). **Table S10.** The serial mediation models in girls in overweight or obese participants (*n* = 86).

## Data Availability

The dataset used and analyzed during the current study is available from the corresponding author on reasonable request.

## Abbreviations

BMI: Body mass index
COTF: Group of China Obesity Task Force
POTS: Perception of Teasing Scale
PSS-10: 10-item Perceived Stress Scale
TFEQ-R18: Three-Factor Eating Questionnaire-Revised 18-item version

## References

[1] Major B, O'Brien LT (2005). The social psychology of stigma. Annu Rev Psychol.

[2] Puhl RM, Latner JD (2007). Stigma, obesity, and the health of the nation’s children. Psychol Bull.

[3] Puhl RM, Latner JD, O'Brien K, Luedicke J, Danielsdottir S, Forhan M (2015). A multinational examination of weight bias: predictors of anti-fat attitudes across four countries. Int J Obes (2005).

[4] Puhl RM, Heuer CA (2009). The stigma of obesity: a review and update. Obesity (Silver Spring, Md).

[5] Puhl RM, Peterson JL, Luedicke J (2013). Weight-based victimization: bullying experiences of weight loss treatment-seeking youth. Pediatrics.

[6] Carr D, Friedman MA (2005). Is obesity stigmatizing? Body weight, perceived discrimination, and psychological well-being in the United States. J Health Soc Behav.

[7] Vartanian LR, Shaprow JG (2008). Effects of weight stigma on exercise motivation and behavior: a preliminary investigation among college-aged females. J Health Psychol.

[8] Brixval CS, Rayce SL, Rasmussen M, Holstein BE, Due P (2012). Overweight, body image and bullying--an epidemiological study of 11- to 15-years olds. Eur J Pub Health.

[9] Andreyeva T, Puhl RM, Brownell KD (2008). Changes in perceived weight discrimination among Americans, 1995–1996 through 2004–2006. Obesity (Silver Spring, Md).

[10] Puhl RM, Heuer CA (2010). Obesity stigma: important considerations for public health. Am J Public Health.

[11] Wellman JD, Araiza AM, Solano C, Berru E (2018). Sex differences in the relationships among weight stigma, depression, and binge eating. Appetite.

[12] Bucchianeri MM, Eisenberg ME, Wall MM, Piran N, Neumark-Sztainer D (2014). Multiple types of harassment: associations with emotional well-being and unhealthy behaviors in adolescents. J Adolesc Health.

[13] Goldfield G, Moore C, Henderson K, Buchholz A, Obeid N, Flament M (2010). The relation between weight-based teasing and psychological adjustment in adolescents. Paediatr Child Health.

[14] Haines J, Neumark-Sztainer D, Eisenberg ME, Hannan PJ (2006). Weight teasing and disordered eating behaviors in adolescents: longitudinal findings from Project EAT (Eating Among Teens). Pediatrics.

[15] Olvera N, Dempsey A, Gonzalez E, Abrahamson C (2013). Weight-related teasing, emotional eating, and weight control behaviors in Hispanic and African American girls. Eat Behav.

[16] Losekam S, Goetzky B, Kraeling S, Rief W, Hilbert A (2010). Physical activity in normal-weight and overweight youth: associations with weight teasing and self-efficacy. Obes Facts.

[17] Jackson SE, Beeken RJ, Wardle J (2014). Perceived weight discrimination and changes in weight, waist circumference, and weight status. Obesity (Silver Spring, Md).

[18] Pont SJ, Puhl R, Cook SR, Slusser W (2017). Stigma experienced by children and adolescents with obesity. Pediatrics.

[19] Cramer P, Steinwert T (1998). Thin is good, fat is bad: how early does it begin?. J Appl Dev Psychol.

[20] Puhl RM, Luedicke J, Heuer C (2011). Weight-based victimization toward overweight adolescents: observations and reactions of peers. J School Health.

[21] Bucchianeri MM, Eisenberg ME, Neumark-Sztainer D (2013). Weightism, racism, classism, and sexism: shared forms of harassment in adolescents. J Adolesc Health.

[22] Puhl RM, Luedicke J (2012). Weight-based victimization among adolescents in the school setting: emotional reactions and coping behaviors. J Youth Adolesc.

[23] Chisuwa-Hayami N, Haruki T (2017). Associations of body-related teasing with weight status, body image, and dieting behavior among Japanese adolescents. Health Promot Perspect.

[24] Tomiyama AJ (2019). Stress and obesity. Annu Rev Psychol.

[25] O'Brien KS, Latner JD, Puhl RM, Vartanian LR, Giles C, Griva K (2016). The relationship between weight stigma and eating behavior is explained by weight bias internalization and psychological distress. Appetite.

[26] Salwen JK, Hymowitz GF, Bannon SM, O'Leary KD (2015). Weight-related abuse: perceived emotional impact and the effect on disordered eating. Child Abuse Negl.

[27] COTF (2004). Body mass index reference norm for screening overweight and obesity in Chinese children and adolescents. Chin J Epidemiol.

[28] Thompson JK, Cattarin J, Fowler B, Fisher E (1995). The perception of teasing scale (POTS): a revision and extension of the physical appearance related teasing scale (PARTS). J Pers Assess.

[29] Wong WKW (1998). Psychosocial adjustment of obese Chinese adolescent girls in Hong Kong.

[30] Angle S, Engblom J, Eriksson T, Kautiainen S, Saha MT, Lindfors P (2009). Three factor eating questionnaire-R18 as a measure of cognitive restraint, uncontrolled eating and emotional eating in a sample of young Finnish females. Int J Behav Nutr Phys Act.

[31] Cheng MY, Wang SM, Lam YY, Luk HT, Man YC, Lin CY (2018). The relationships between weight Bias, perceived weight stigma, eating behavior, and psychological distress among undergraduate students in Hong Kong. J Nerv Ment Dis.

[32] Cohen S, Williamson GM (1991). Stress and infectious disease in humans. Psychol Bull.

[33] Lu W, Bian Q, Wang W, Wu X, Wang Z, Zhao M (2017). Chinese version of the perceived stress scale-10: a psychometric study in Chinese university students. PLoS One.

[34] Hayes AF (2013). Introduction to mediation, moderation, and conditional process analysis: a regression-based approach.

[35] IBM Corp. IBM SPSS Statistics for Windows, Version 22.0. Armonk: IBM Corp; 2013.

[36] Durso LE, Latner JD, Hayashi K (2012). Perceived discrimination is associated with binge eating in a community sample of non-overweight, overweight, and obese adults. Obes Facts.

[37] Culbert KM, Racine SE, Klump KL (2015). Research review: what we have learned about the causes of eating disorders - a synthesis of sociocultural, psychological, and biological research. J Child Psychol Psychiatry.

[38] Field AE, Camargo CA, Taylor CB, Berkey CS, Roberts SB, Colditz GA (2001). Peer, parent, and media influences on the development of weight concerns and frequent dieting among preadolescent and adolescent girls and boys. Pediatrics.

[39] Homan K (2010). Athletic-ideal and thin-ideal internalization as prospective predictors of body dissatisfaction, dieting, and compulsive exercise. Body Image.

[40] Hunger JM, Tomiyama AJ (2018). Weight labeling and disordered eating among adolescent girls: longitudinal evidence from the National Heart, Lung, and Blood Institute growth and health study. J Adolesc Health.

[41] Puhl RM, Wall MM, Chen C, Bryn Austin S, Eisenberg ME, Neumark-Sztainer D (2017). Experiences of weight teasing in adolescence and weight-related outcomes in adulthood: a 15-year longitudinal study. Prev Med.

[42] Vartanian LR, Porter AM (2016). Weight stigma and eating behavior: a review of the literature. Appetite.

[43] Crowther JH, Sanftner J, Bonifazi DZ, Shepherd KL (2001). The role of daily hassles in binge eating. Int J Eat Disord.

[44] Gan WY, Mohd Nasir MT, Zalilah MS, Hazizi AS (2012). Psychological distress as a mediator in the relationships between biopsychosocial factors and disordered eating among Malaysian university students. Appetite.

[45] Hayward LE, Vartanian LR, Pinkus RT (2018). Weight stigma predicts poorer psychological well-being through internalized weight bias and maladaptive coping responses. Obesity (Silver Spring, Md).

[46] Phelan SM, Burgess DJ, Puhl R, Dyrbye LN, Dovidio JF, Yeazel M (2015). The adverse effect of weight stigma on the well-being of medical students with overweight or obesity: findings from a national survey. J Gen Intern Med.

[47] Demetriou C, Ozer BU, Essau CA (2015). Self-report questionnaires. The encyclopedia of clinical psychology.

