# Supplementary material for: Relationships among weight stigma, eating behaviors and stress in adolescents in Wuhan, China
Source: Glob Health Res Policy. 2020 Mar 7;5:8. doi: 10.1186/s41256-020-00138-3 (PMC7060634; doi:10.1186/s41256-020-00138-3)
Supplement: Supplementary file 1 — Additional file 1: Table S1. Correlations between all study variables. Correlations for boys in non-overweight participants (n = 742) and girls in non-overweight participants (n = 745), respectively. Table S2. Correlations between all study variables. Correlations for boys in overweight or obese participants (n = 742) and girls in overweight or obese participants (n = 745), respectively. Table S3. Hierarchical linear regression analyses in boys in non-overweight participants (n = 742). Table S4. Hierarchical linear regression analyses in girls in non-overweight participants (n = 745). Table S5. Hierarchical linear regression analyses in boys in overweight or obese participants (n = 245). Table S6. Hierarchical linear regression analyses in girls in overweight or obese participants (n = 86). Table S7. The serial mediation models in boys in non-overweight participants (n = 742). Table S8. The serial mediation models in girls in non-overweight participants (n = 745). Table S9. The serial mediation models in boys in overweight or obese participants (n = 245). Table S10. The serial mediation models in girls in overweight or obese participants (n = 86). [file 41256_2020_138_MOESM1_ESM.docx]

**Table S1.** Correlations between all study variables.

|  | **1** | **2** | **3** | **4** | **5** | **6** |
| --- | --- | --- | --- | --- | --- | --- |
| 1. **Age** | - | 0.076* | -0.036 | -0.089* | -0.008 | 0.040 |
| 1. **Stigma** | 0.074* | - | 0.206*** | 0.171*** | 0.208*** | 0.156*** |
| 1. **Cognitive restraint** | -0.016 | 0.191*** | - | 0.087* | 0.127** | 0.039 |
| 1. **Uncontrolled eating** | -0.054 | 0.242*** | -0.120** | - | 0.605*** | 0.213*** |
| 1. **Emotional eating** | 0.067 | 0.271*** | -0.001 | 0.611*** | - | 0.180*** |
| 1. **Perceived stress** | 0.005 | 0.325*** | 0.019 | 0.302*** | 0.273*** | - |

Notes: Correlations for boys in non-overweight participants (n = 742) are displayed above the diagonal, and girls (n = 745) are displayed below the diagonal.

*p < 0.05, **p < 0.01, ***p < 0.001.

**Table S2.** Correlations between all study variables.

|  | **1** | **2** | **3** | **4** | **5** | **6** |
| --- | --- | --- | --- | --- | --- | --- |
| 1. **Age** | - | -0.050 | 0.074 | -0.193** | -0.045 | -0.074 |
| 1. **Stigma** | -0.097 | - | 0.047 | 0.350*** | 0.301*** | 0.275*** |
| 1. **Cognitive restraint** | 0.059 | -0.072 | - | -0.128* | -0.039 | -0.054 |
| 1. **Uncontrolled eating** | -0.100 | 0.258* | -0.337** | - | 0.632*** | 0.163* |
| 1. **Emotional eating** | -0.014 | 0.297** | -0.194 | 0.586*** | - | 0.166** |
| 1. **Perceived stress** | 0.056 | 0.401*** | 0.023 | 0.311** | 0.264* | - |

Notes: Correlations for boys in overweight/obese participants (n = 245) are displayed above the diagonal, and girls (n = 86) are displayed below the diagonal.

*p < 0.05, **p < 0.01, ***p < 0.001.

**Table S3.** Hierarchical linear regression analyses in boys in non-overweight participants (n = 742).

| **Variables** | **Model1** | | | **Model2** | | | **Model3** | | |
| --- | --- | --- | --- | --- | --- | --- | --- | --- | --- |
|  | **B** | **SE** | **β** | **B** | **SE** | **β** | **B** | **SE** | **β** |
| Cognitive restraint |  |  |  |  |  |  |  |  |  |
| Age | -0.153 | 0.157 | -0.036 | -0.221 | 0.154 | -0.052 | -0.222 | .154 | -.052 |
| Stigma |  |  |  | 0.125 | 0.021 | 0.210*** | 0.124 | 0.022 | 0.208*** |
| Stress |  |  |  |  |  |  | 0.005 | 0.022 | 0.009 |
| R^2^ |  |  | 0.001 |  |  | 0.045*** |  |  | 0.045 |
| Uncontrolled eating |  |  |  |  |  |  |  |  |  |
| Age | -0.481 | 0.199 | -0.089* | -0.555 | 0.197 | -0.102** | -0.585 | 0.193 | -0.107** |
| Stigma |  |  |  | 0.135 | 0.027 | 0.178*** | 0.113 | 0.027 | 0.148*** |
| Stress |  |  |  |  |  |  | 0.150 | 0.028 | 0.194*** |
| R^2^ |  |  | 0.008* |  |  | 0.039 *** |  |  | 0.076*** |
| Emotional eating |  |  |  |  |  |  |  |  |  |
| Age | -0.019 | 0.089 | -0.008 | -0.058 | 0.087 | -0.024 | -0.068 | 0.086 | -0.028 |
| Stigma |  |  |  | 0.071 | 0.012 | 0.210*** | 0.063 | 0.012 | 0.187*** |
| Stress |  |  |  |  |  |  | 0.052 | 0.012 | 0.152*** |
| R^2^ |  |  | 0.000 |  |  | 0.044*** |  |  | 0.066*** |

B Unstandardized coefficients, SE standard errors, β standardized coefficients.

*p < 0.05, **p < 0.01, ***p < 0.001.

**Table S4.** Hierarchical linear regression analyses in girls in non-overweight participants (n = 745).

| **Variables** | **Model1** | | | **Model2** | | | **Model3** | | |
| --- | --- | --- | --- | --- | --- | --- | --- | --- | --- |
|  | **B** | **SE** | **β** | **B** | **SE** | **β** | **B** | **SE** | **β** |
| Cognitive restraint |  |  |  |  |  |  |  |  |  |
| Age | -0.060 | 0.140 | -0.016 | -0.114 | 0.137 | -0.030 | -0.118 | 0.137 | -0.031 |
| Stigma |  |  |  | 0.080 | 0.015 | 0.193*** | 0.087 | 0.016 | 0.209*** |
| Stress |  |  |  |  |  |  | -0.028 | 0.022 | -0.049 |
| R^2^ |  |  | 0.000 |  |  | 0.037*** |  |  | 0.039 |
| Uncontrolled eating |  |  |  |  |  |  |  |  |  |
| Age | -0.291 | 0.196 | -0.054 | -0.390 | 0.191 | -0.073* | -0.364 | 0.185 | -0.068 |
| Stigma |  |  |  | 0.145 | 0.021 | 0.247*** | 0.097 | 0.021 | 0.166*** |
| Stress |  |  |  |  |  |  | 0.199 | 0.029 | 0.249*** |
| R^2^ |  |  | 0.003 |  |  | 0.064*** |  |  | 0.119*** |
| Emotional eating |  |  |  |  |  |  |  |  |  |
| Age | 0.175 | 0.095 | 0.067 | 0.123 | 0.092 | 0.047 | 0.134 | 0.090 | 0.051 |
| Stigma |  |  |  | 0.076 | 0.010 | 0.267*** | 0.056 | 0.010 | 0.199*** |
| Stress |  |  |  |  |  |  | 0.081 | 0.014 | 0.208*** |
| R^2^ |  |  | 0.005 |  |  | 0.075*** |  |  | 0.114*** |

B Unstandardized coefficients, SE standard errors, β standardized coefficients.

*p < 0.05, **p < 0.01, ***p < 0.001.

**Table S5.** Hierarchical linear regression analyses in boys in overweight or obese participants (n = 245).

| **Variables** | **Model1** | | | **Model2** | | | **Model3** | | |
| --- | --- | --- | --- | --- | --- | --- | --- | --- | --- |
|  | **B** | **SE** | **β** | **B** | **SE** | **β** | **B** | **SE** | **β** |
| Cognitive restraint |  |  |  |  |  |  |  |  |  |
| Age | 0.296 | 0.257 | 0.074 | 0.306 | 0.258 | 0.076 | 0.290 | 0.258 | 0.072 |
| Stigma |  |  |  | 0.018 | 0.023 | 0.051 | 0.024 | 0.023 | 0.069 |
| Stress |  |  |  |  |  |  | -0.037 | 0.037 | -0.067 |
| R^2^ |  |  | 0.005 |  |  | 0.008 |  |  | 0.012 |
| Uncontrolled eating |  |  |  |  |  |  |  |  |  |
| Age | -1.261 | 0.411 | -0.193** | -1.148 | 0.386 | -0.176** | -1.124 | 0.387 | -0.172** |
| Stigma |  |  |  | 0.195 | 0.034 | 0.341*** | 0.185 | 0.035 | 0.325*** |
| Stress |  |  |  |  |  |  | 0.055 | 0.055 | 0.061 |
| R^2^ |  |  | 0.037** |  |  | 0.153 *** |  |  | 0.157 |
| Emotional eating |  |  |  |  |  |  |  |  |  |
| Age | -0.138 | 0.194 | -0.045 | -0.092 | 0.186 | -0.030 | -0.076 | 0.186 | -0.025 |
| Stigma |  |  |  | 0.080 | 0.016 | 0.300*** | 0.073 | 0.017 | 0.276*** |
| Stress |  |  |  |  |  |  | 0.037 | 0.026 | 0.088 |
| R^2^ |  |  | 0.002 |  |  | 0.092*** |  |  | 0.099 |

B Unstandardized coefficients, SE standard errors, β standardized coefficients.

*p < 0.05, **p < 0.01, ***p < 0.001.

**Table S6.** Hierarchical linear regression analyses in girls in overweight or obese participants (n = 86).

| **Variables** | **Model1** | | | **Model2** | | | **Model3** | | |
| --- | --- | --- | --- | --- | --- | --- | --- | --- | --- |
|  | **B** | **SE** | **β** | **B** | **SE** | **β** | **B** | **SE** | **β** |
| Cognitive restraint |  |  |  |  |  |  |  |  |  |
| Age | 0.240 | 0.442 | 0.059 | 0.213 | 0.446 | 0.053 | 0.191 | 0.450 | 0.047 |
| Stigma |  |  |  | -0.023 | 0.038 | -0.067 | -0.031 | 0.042 | -0.090 |
| Stress |  |  |  |  |  |  | 0.028 | 0.061 | 0.056 |
| R^2^ |  |  | 0.003 |  |  | 0.008 |  |  | 0.011 |
| Uncontrolled eating |  |  |  |  |  |  |  |  |  |
| Age | -0.563 | 0.611 | -0.100 | -0.426 | 0.598 | -0.076 | -0.566 | 0.586 | -0.101 |
| Stigma |  |  |  | 0.120 | 0.051 | 0.251* | 0.069 | 0.054 | 0.144 |
| Stress |  |  |  |  |  |  | 0.181 | 0.079 | 0.259* |
| R^2^ |  |  | 0.010 |  |  | 0.072* |  |  | 0.128* |
| Emotional eating |  |  |  |  |  |  |  |  |  |
| Age | -0.042 | 0.320 | -0.014 | 0.043 | 0.309 | 0.015 | -0.005 | 0.308 | -0.002 |
| Stigma |  |  |  | 0.074 | 0.026 | 0.298** | 0.057 | 0.029 | 0.228 |
| Stress |  |  |  |  |  |  | 0.063 | 0.042 | 0.173 |
| R^2^ |  |  | 0.000 |  |  | 0.088** |  |  | 0.113 |

B Unstandardized coefficients, SE standard errors, β standardized coefficients.

*p < 0.05, **p < 0.01, ***p < 0.001.

**Table S7.** The serial mediation models in boys in non-overweight adolescents (n = 742).

| **Antecedent** | **Consequent** | | | | | | | |
| --- | --- | --- | --- | --- | --- | --- | --- | --- |
|  |  | **Stress** | | |  | **Cognitive restraint** | | |
|  |  | **coeff.** | **SE** | **P-Value** |  | **coeff.** | **SE** | **P-Value** |
| Stigma | a_1_ | 0.1516 | 0.0359 | 0.0000 | c’ | 0.1240 | 0.0217 | 0.0000 |
| Stress |  | - | - | - | b_1_ | 0.0054 | 0.0220 | 0.8052 |
| Antecedent |  | Stress | | |  | Uncontrolled eating | | |
|  |  | coeff. | SE | P |  | coeff. | SE | P |
| Stigma | a_1_ | 0.1516 | 0.0359 | 0.0004 | c’ | 0.1128 | 0.0273 | 0.0000 |
| Stress |  | - | - | - | b_1_ | 0.1496 | 0.0277 | 0.0000 |
| Antecedent |  | Stress | | |  | Emotional eating | | |
|  |  | coeff. | SE | P |  | coeff. | SE | p |
| Stigma | a_1_ | 0.1516 | 0.0359 | 0.0004 | c’ | 0.0628 | 0.0122 | 0.0000 |
| Stress |  | - | - | - | b_1_ | 0.0518 | 0.0123 | 0.0000 |

coeff. Regression coefficients, SE standard errors.

*Statistically significant, p < 0.05.

**Table S8.** The serial mediation models in girls in non-overweight adolescents (n = 745).

| **Antecedent** | **Consequent** | | | | | | | |
| --- | --- | --- | --- | --- | --- | --- | --- | --- |
|  |  | **Stress** | | |  | **Cognitive restraint** | | |
|  |  | **coeff.** | **SE** | **P-Value** |  | **coeff.** | **SE** | **P-Value** |
| Stigma | a_1_ | 0.2382 | 0.0254 | 0.0000 | c’ | 0.0867 | 0.0158 | 0.0000 |
| Stress |  | - | - | - | b_1_ | -0.0279 | 0.0216 | 0.1972 |
| Antecedent |  | Stress | | |  | Uncontrolled eating | | |
|  |  | coeff. | SE | p |  | coeff. | SE | p |
| Stigma | a_1_ | 0.2382 | 0.0254 | 0.0000 | c’ | 0.0971 | 0.0214 | 0.0000 |
| Stress |  | - | - | - | b_1_ | 0.1994 | 0.0292 | 0.0000 |
| Antecedent |  | Stress | | |  | Emotional eating | | |
|  |  | coeff. | SE | p |  | coeff. | SE | p |
| Stigma total | a_1_ | 0.2382 | 0.0254 | 0.0000 | c’ | 0.0565 | 0.0104 | 0.0000 |
| Stress |  | - | - | - | b_1_ | 0.0808 | 0.0142 | 0.0000 |

coeff. Regression coefficients, SE standard errors.

*Statistically significant, p < 0.05.

**Table S9.** The serial mediation models in boys in overweight or obese adolescents (n = 245).

| **Antecedent** | **Consequent** | | | | | | | |
| --- | --- | --- | --- | --- | --- | --- | --- | --- |
|  |  | **Stress** | | |  | **Cognitive restraint** | | |
|  |  | **coeff.** | **SE** | **P-Value** |  | **coeff.** | **SE** | **P-Value** |
| Stigma | a_1_ | 0.1735 | 0.0394 | 0.0000 | c’ | 0.0243 | 0.0235 | 0.3020 |
| Stress |  | - | - | - | b_1_ | -0.0372 | 0.0368 | 0.3130 |
| Antecedent |  | Stress | | |  | Uncontrolled eating | | |
|  |  | coeff. | SE | P |  | coeff. | SE | P |
| Stigma | a_1_ | 0.1735 | 0.0394 | 0.0000 | c’ | 0.1855 | 0.0352 | 0.0000 |
| Stress |  | - | - | - | b_1_ | 0.0546 | 0.0552 | 0.3236 |
| Antecedent |  | Stress | | |  | Emotional eating | | |
|  |  | coeff. | SE | P |  | coeff. | SE | p |
| Stigma total | a_1_ | 0.1735 | 0.0394 | 0.0000 | c’ | 0.0733 | 0.0169 | 0.0000 |
| Stress |  | - | - | - | b_1_ | 0.0365 | 0.0265 | 0.1691 |

coeff. Regression coefficients, SE standard errors.

*Statistically significant, p < 0.05.

**Table S10.** The serial mediation models in girls in overweight or obese adolescents (n = 86).

| **Antecedent** | **Consequent** | | | | | | | |
| --- | --- | --- | --- | --- | --- | --- | --- | --- |
|  |  | **Stress** | | |  | **Cognitive restraint** | | |
|  |  | **coeff.** | **SE** | **P-Value** |  | **coeff.** | **SE** | **P-Value** |
| Stigma | a_1_ | 0.2808 | 0.0688 | 0.0001 | c’ | -0.0311 | 0.0417 | 0.4577 |
| Stress |  | - | - | - | b_1_ | 0.0282 | 0.0607 | 0.6433 |
| Antecedent |  | Stress | | |  | Uncontrolled eating | | |
|  |  | coeff. | SE | p |  | coeff. | SE | p |
| Stigma | a_1_ | 0.2808 | 0.0688 | 0.0001 | c’ | 0.0690 | 0.0543 | 0.2072 |
| Stress |  | - | - | - | b_1_ | 0.1810 | 0.0790 | 0.0246 |
| Antecedent |  | Stress | | |  | Emotional eating | | |
|  |  | coeff. | SE | p |  | coeff. | SE | p |
| Stigma total | a_1_ | 0.2808 | 0.0688 | 0.0001 | c’ | 0.0567 | 0.0285 | 0.0500 |
| Stress |  | - | - | - | b_1_ | 0.0628 | 0.0415 | 0.1345 |

coeff. Regression coefficients, SE standard errors.

*Statistically significant, p < 0.05.
